# The erythropoietin receptor expressed in skeletal muscle is essential for mitochondrial biogenesis and physiological exercise

**DOI:** 10.1007/s00424-021-02577-4

**Published:** 2021-06-17

**Authors:** Kirsten T. Nijholt, Laura M. G. Meems, Willem P. T. Ruifrok, Alexander H. Maass, Salva R. Yurista, Mario G. Pavez-Giani, Belend Mahmoud, Anouk H. G. Wolters, Dirk J. van Veldhuisen, Wiek H. van Gilst, Herman H. W. Silljé, Rudolf A. de Boer, B. Daan Westenbrink

**Affiliations:** 1 Department of Cardiology, University Medical Centre Groningen, University of Groningen, HPC AB31, 9700 RB, P.O. Box 30.001 Groningen, The Netherlands; 2 Department of Cell Biology, University Medical Centre Groningen, University of Groningen, Groningen, The Netherlands

**Keywords:** Erythropoietin receptor, Cardiac and skeletal muscle, Mitochondrial biogenesis, Exercise performance, Exercise-induced physiological adaptation

## Abstract

Erythropoietin (EPO) is a haematopoietic hormone that regulates erythropoiesis, but the EPO-receptor (EpoR) is also expressed in non-haematopoietic tissues. Stimulation of the EpoR in cardiac and skeletal muscle provides protection from various forms of pathological stress, but its relevance for normal muscle physiology remains unclear. We aimed to determine the contribution of the tissue-specific EpoR to exercise-induced remodelling of cardiac and skeletal muscle. Baseline phenotyping was performed on left ventricle and m. gastrocnemius of mice that only express the EpoR in haematopoietic tissues (EpoR-tKO). Subsequently, mice were caged in the presence or absence of a running wheel for 4 weeks and exercise performance, cardiac function and histological and molecular markers for physiological adaptation were assessed. While gross morphology of both muscles was normal in EpoR-tKO mice, mitochondrial content in skeletal muscle was decreased by 50%, associated with similar reductions in mitochondrial biogenesis, while mitophagy was unaltered. When subjected to exercise, EpoR-tKO mice ran slower and covered less distance than wild-type (WT) mice (5.5 ± 0.6 vs. 8.0 ± 0.4 km/day, p < 0.01). The impaired exercise performance was paralleled by reductions in myocyte growth and angiogenesis in both muscle types. Our findings indicate that the endogenous EPO-EpoR system controls mitochondrial biogenesis in skeletal muscle. The reductions in mitochondrial content were associated with reduced exercise capacity in response to voluntary exercise, supporting a critical role for the extra-haematopoietic EpoR in exercise performance.

**Supplementary Information:**

The online version contains supplementary material available at 10.1007/s00424-021-02577-4.

## Introduction

Erythropoietin (EPO) is a hormone that is secreted by the adult kidneys and the foetal liver and regulates erythropoiesis in the haematopoietic tissues [46, 61]. EPO stimulates erythropoiesis by binding to a homodimeric EPO-receptor (EpoR) on erythroid progenitor cells, which promotes their survival, proliferation and differentiation into erythrocytes [46, 61]. In certain pathological conditions such as chronic renal failure, EPO production becomes insufficient and anaemia ensues [46, 54, 61]. Treatment with recombinant human EPO (rhEPO) does not only increase haematocrit in these patients, but also improves quality of life and promotes exercise capacity [9, 31]. rhEPO is also used to enhance exercise performance by various endurance athletes without anaemia [3, 4].

The mechanisms underlying the effects of rhEPO on exercise are generally thought to result from the associated increase in haematocrit, under the assumption that this augments oxygen delivery to aerobic tissues [4, 25–27]. Although this may partially explain the beneficial effects in patients with severe anaemia, extra-haematopoietic effects may also contribute to the improvements in quality of life and exercise performance [14, 25]. For instance, studies have shown that rhEPO activates mitochondrial biogenesis and subsequently promotes exercise capacity, prior to an elevation in haematocrit [7]. Also, enhancement of mitochondrial function was observed in skeletal muscle fibres of athletes after rhEPO administration [39]. Indeed, most of the effects of rhEPO on exercise performance in endurance athletes are not exclusively explained by changes in haematocrit and the accessory effects could therefore result from non-haematopoietic effects [2].

There are several lines of evidence supporting the concept that EPO augments exercise performance by activating an EpoR subtype in non-haematopoietic tissues [36]. This EpoR has been discovered in a variety of non-haematopoietic tissues, including cardiac and skeletal muscle, endothelial cells, neurones and kidneys [34, 36, 46]. Evidence for its role in tissue physiology is based upon studies where rhEPO protected these tissues from various forms of pathological stress [10, 12, 24, 55, 56], even when administered in doses that are too low to stimulate erythropoiesis [23]. More direct evidence for the significance of the extra-haematopoietic EPO-EpoR system comes from studies with a mouse model that only expresses the EpoR in haematopoietic cells, but lack EpoR expression in all other tissues (EpoR-tissue knock-out, EpoR-tKO mice) [47]. While these EpoR-tKO mice display a normal phenotype with preserved erythropoiesis, their non-haematopoietic tissues are more vulnerable to pathological stress [10, 50]. For instance, cardiac and skeletal muscle of EpoR-tKO mice are more susceptible to ischemic or toxic injury and also develop more severe heart failure when subjected to pressure overload [16, 33, 48].

While this evidence indicates that the EpoR expressed in muscle is critical for the adaptation to pathological stress, its relevance for muscle physiology is not well described. We hypothesised that the EpoR expressed in cardiac and skeletal muscle is critical for the physiological adaptation to exercise. To test this hypothesis, we subjected EpoR-tKO mice to a model of voluntary wheel running.

## Methods

## Ethical approval

The experimental protocol was accepted by The Animal Ethical Committee of the University of Groningen (DEC4585, IvD 1583–02-011, 1583–02-014). In addition, all animal experiments followed the protocols from Directive 2010/63/EU of the European Parliament.

### Mouse model

**Fig. 1:**
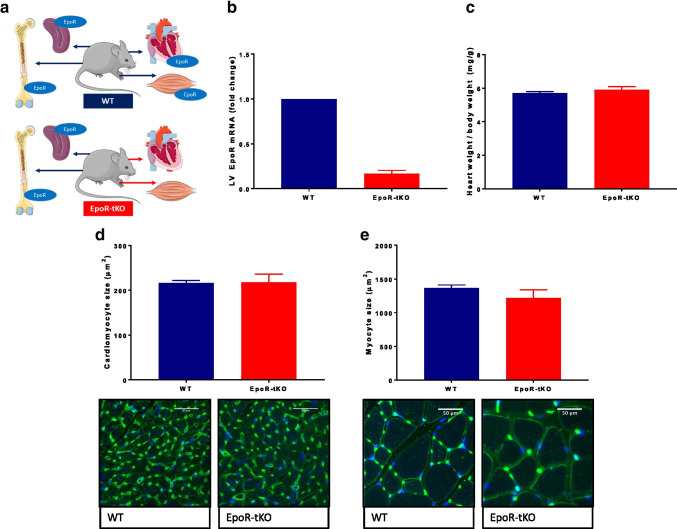
Baseline muscle phenotype of tissue-specific erythropoietin receptor deficient mice. Phenotyping of tissue-specific erythropoietin receptor deficient (EpoR-tKO) mice. Shown are (**a**) conceptual illustration of the mouse model, in which the erythropoietin receptor (EpoR) is absent in non-haematopoietic tissues of EpoR-tKO mice, (**b**) left ventricular (LV) mRNA expression of the EpoR, (n = 2–5/group), (**c**) heart weight to body weight ratio in milligrams per gram (mg/g), (**d**) cardiomyocyte cross-sectional area in micrometres squared (μm^2^) and typical examples, and (**e**) skeletal myocyte cross-sectional area (μm^2^) including typical examples, (n = 11–13/group). WT wild-type mice, EpoR-tKO EpoR-tissue knock-out mice. Data are presented as mean value ± standard error of the mean (SEM). The illustration in panel **a** contains images from Servier Medical Art by Servier, licensed under a Creative Commons Attribution 3.0 unported license

We employed the well-described EpoR-/-rescued mouse model in which EpoR-tissue knock-out mice have been rescued from lethality by a transgene that drives EpoR expression exclusively in haematopoietic tissues [47]. These mice (EpoR-tissue knock-out; EpoR-tKO) develop normally and are fertile without a clear (cardiac) phenotype, despite the absence of the EPO receptor in all non-haematopoietic tissues (Fig. 1) [47]. EpoR-tKO mice originated from the Riken BioResource Research Center (Riken BRC) repository in Japan. Thereafter, the EpoR-tKO mice were bred and housed at the central animal facility of the University of Groningen and back-crossed into our local C57Bl/6 J genetic background. Age and gender-matched wild-type (WT) mice were used as controls. Genotyping of EpoR-tKO mice was performed as described previously [47].

### Experimental model

At 8–12 weeks of age, WT and EpoR-tKO male mice were randomised to the presence or absence of a running wheel present in their individual cage. The voluntary running wheel (VWR) was present for the whole duration of the experiment, which was a period of 4 weeks (referred to as WT Run or EpoR-tKO Run). Control littermates were housed similarly but without a running wheel and therefore remained sedentary for this period (referred to as WT Sed or EpoR-tKO Sed). An additional group of male mice was selected for phenotyping of basal mitochondrial parameters. In total, 89 male mice were used for this study. All mice had ad libitum access to food and water.

### Exercise performance

A cyclometer was attached to the running wheel, which measured daily exercise parameters, including speed, distance and time on the running wheel, as described previously [1].

### Cardiac function parameters and haemodynamics

Following completion of the 4-week experiment and 2 days prior to sacrifice, cardiac function was determined by echocardiography (GE Vivid 7 Dimension, using a 13-MHz probe) as described before [29]. Subsequently, on the day of sacrifice, haemodynamic parameters were determined with a microtip pressure–volume transducer as described previously (Millar Instr. Inc., Houston, TX, USA) [37]. All cardiac function assessments were performed under general anaesthesia (isoflurane 2%).

### Organ, body and blood measurements

After haemodynamic measurements, blood was drawn and hearts plus skeletal muscle were collected. After weighing the hearts, the left ventricle (LV) and the gastrocnemius skeletal muscle were fixated for immunohistochemistry or snap frozen for molecular analysis.

### Histology

After isolation of LV and m. gastrocnemius, cardiac muscle was cut at the mid-papillary level, and skeletal muscle was cut at the mid-belly level. Sections were either fixated in 4% paraformaldehyde and embedded in paraffin after dehydration steps (Leica TP1020, Germany) or fixated in Tissue-Tec and subsequently slowly frozen in liquid nitrogen. Following fixation and embedding processes, sections were cut using a microtome and were thereafter processed onto slides.

#### (Cardio) Myocyte size

To determine (cardio) myocyte cross sectional area, deparaffinised 3-μm thick sections were stained with 4′,6-diamidino-2-phenylindole (DAPI) (Vector Laboratories, USA) and wheat germ agglutinin (WGA) (Sigma-Aldrich, USA), as described before [60]. Stained transverse sections were visualised with fluorescence microscopy to generate images for analysis (Zeiss KS400, Germany). Quantification fields were chosen at random at × 40 magnification; five fields per sample were counted and averaged.

#### Capillary density

To measure capillary density as a measure of adaptive angiogenesis, endothelial cells were incubated with the endothelial cell antigen CD31 (purified rat anti-mouse CD31 antibody #550,274, BD Biosciences, USA). Prior to incubation of antibodies, cryosections were fixated in acetone, air-dried and blocked for endogenous peroxidases. After primary, secondary (unconjugated rabbit anti-rat IgG antibody, #AI-4001, Vector Laboratories, USA) and tertiary (anti-rabbit polymer-HRP, #K4008, EnVision kit, Denmark) antibody incubation, AEC + solution was applied and counterstained with Mayer’s hematoxylin. Capillaries were quantified from at least eight randomly chosen fields at × 40 magnification (Zeiss KS400, Germany), and adaptive angiogenesis was presented as capillary to (cardio)myocyte ratio [5].

### Electron microscopy

To assess basal mitochondrial quantity, large-scale electron microscopy (EM), known as nanotomy (for nano-anatomy), was performed. Samples from skeletal muscle were cut into pieces of approximately 1 mm^2^ and were fixated in 2% glutaraldehyde and 2% paraformaldehyde solution in 0.1 M sodium cacodylate directly after sacrifice. Following fixation, samples were further processed with 1% osmium tetroxide and 1.5% potassium ferrocyanide and, finally, after dehydration embedded in EPON epoxy resin. Sections of 80 nm were cut using an ultramicrotome, which was followed by contrast staining with 4% neodymium acetate for 30 min [19]. Finally, sections were observed using scanning and transmission electron microscopy (STEM) (Zeiss Supra55, Oberkochen Germany), as described before [15, 18, 45]. This microscope was connected to an external scan generator ATLAS5 (Fibics, Canada) to produce many high-resolution images that are stitched to generate a large Nanotomy ‘map’. TIFF files were converted to html links and uploaded to the website www.nanotomy.org. Of each nanotomy file, at least six high-resolution images, selected randomly in the map, were saved for analysis of mitochondrial quantity. Analysis of images was performed in ImageJ (Fiji- ImageJ version of Java 6, USA).

### Quantitative real-time polymerase chain reaction

To assess molecular analysis at mRNA level, qRT-PCR was performed as described before [58]. Total RNA from LV and m. gastrocnemius was isolated using the Trizol isolation method (Invitrogen Corporation, USA). Quantification of RNA concentrations was performed using Nanodrop (ThermoFisher, USA). cDNA synthesis was performed with equal RNA concentrations (500 ng of total RNA) using the Quantitect Reverse Transcription kit (Qiagen, the Netherlands). qRT-PCR was performed using SYBR Green dye super mix and the running protocol included 3 min at 95 °C, followed by 35 cycles of 15 s at 95 °C, 30 s at 60 °C, followed by dissociation and melting steps (Bio-Rad CFX384, USA). Data were processed according to the ddCT method, and were normalised for housekeeping gene 36B4 and control group WT sedentary. The Online Resource with Supplementary Table S1 contains the primer sequences.

#### Mitochondrial DNA to nuclear DNA ratio (mitochondrial DNA copy number)

To quantify basal mitochondrial DNA copy number as a measure for mitochondrial biogenesis, qRT-PCR was performed for genes encoding mitochondrial proteins. DNA was isolated from LV and m. gastrocnemius using the Nucleospin Tissue XS kit (Macherey–Nagel, Germany); DNA concentrations were quantified using Nanodrop (Thermofisher, USA). A total of 10 μg DNA was used for qRT-PCR, using the standard running protocol as described above. To determine mitochondrial DNA copy number, nuclear DNA-encoded mitochondrial protein hexokinase 2 (HK2) and mitochondrial DNA (mtDNA)-encoded protein NADH dehydrogenase (ND1) were detected [41]. mtDNA copy number was calculated using the ddCT method. HK2 served as reference gene and the WT sedentary group served as control. Supplementary Table S1 contains the primer sequences.

### Western blot

To detect molecular changes at protein level, Western blot was performed [59], specifically to assess levels of the key regulators of mitochondrial biogenesis and mitophagy; nuclear respiratory factor 2 (NRF2), outer mitochondrial membrane protein TOM20 (TOM20), ubiquitin protein 62 (p62) and light chain 3B (LC3B). Total protein was extracted with RIPA buffer and fresh sodium vanadate, phosphatases and proteases buffers (Sigma-Aldrich, USA). Quantification of protein concentrations was performed using the BCA protein assay (Pierce. No. 232250, ThermoFisher, USA). Equal concentrations of protein were boiled and loaded on SDS-PAGE. Semi-dry blotting transferred the proteins onto PVDF membranes. Membranes were incubated with the following primary antibodies: polyclonal anti-NRF2 (ab31163, Abcam, UK), monoclonal anti-TOM20 (#42,406, Cell Signalling, USA), monoclonal anti-sqstm1/p62 antibody (ab56416, Abcam, UK), monoclonal anti-LC3B antibody (#2775, Cell Signalling, USA) and monoclonal anti-GAPDH antibody as a loading control (10R-G109A, Fitzgerald, USA). This was followed by secondary antibody incubation, and proteins were detected with enhanced chemiluminescence (NEL120E001EA, Western lightening ECL Pro, Perkin Elmer, USA) using ImageQuant imager. Western blots were quantified in ImageJ and values were normalised for loading control GAPDH expressions as well as for the control WT sedentary group.

### Statistical analyses

Results are presented as mean value ± standard error of the mean (SEM). Statistical comparisons between two groups with normally distributed data were performed with a student’s t test; if data was not normally distributed, the non-parametric Mann Whitney U test was performed. Statistical comparisons among more than two groups were performed by one-way ANOVA with Bonferroni post hoc test, if distributed normally. Otherwise, the non-parametric Kruskal–Wallis test was used followed by the Mann–Whitney U test. Statistical significance was stated if the p value was < 0.05, of which all p values were two-tailed. Statistical analysis was performed in STATA v11SE software (College Station, TX) and Graph Pad Prism software (Version 7, USA).

## Results

### Phenotyping of basal cardiac and skeletal muscle in tissue-specific erythropoietin receptor-deficient (EpoR-tKO) mice

A conceptual illustration of the mouse model is presented in Fig. 1a. In accordance with this concept, EpoR mRNA expression was undetectable in cardiac muscle from EpoR-tKO mice (Fig. 1b), while haematocrit levels were unaltered, and thus, erythropoiesis was preserved (Supplementary Fig. S1). Cardiac mass and cardiomyocyte cross-sectional area were comparable between EpoR-tKO and WT mice (Fig. 1c–d), as was the mRNA expression of natriuretic peptides and muscle isoforms (Supplementary Fig. S1). Skeletal myocyte size in m. gastrocnemius was also comparable between EpoR-tKO and WT mice (Fig. 1e).

### Basal mitochondrial biogenesis is disrupted in skeletal but not in cardiac muscle of EpoR-tKO mice

**Fig. 2:**
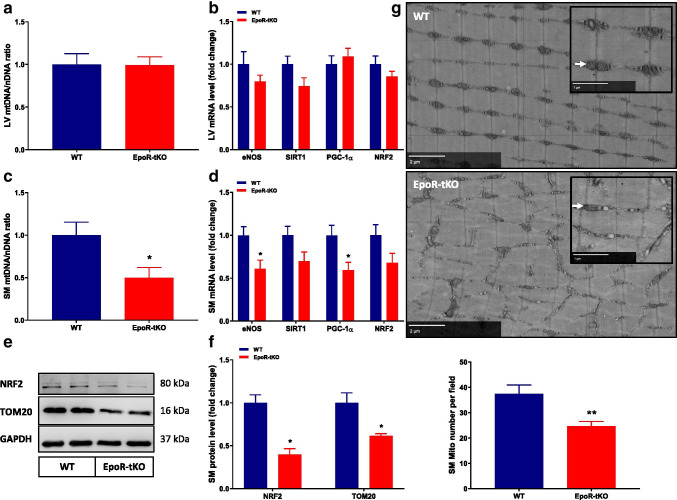
Mitochondrial biogenesis in basal cardiac and skeletal muscle. Cardiac and skeletal muscle mitochondrial biogenesis parameters at baseline are depicted (n = 11–13/group). Shown are (**a**) left ventricular (LV) mitochondrial to nuclear DNA ratio (mtDNA/nDNA), (**b**) LV mRNA expression of markers for mitochondrial biogenesis, (**c**) skeletal muscle (SM) mitochondrial to nuclear DNA ratio (mtDNA/nDNA), (**d**) SM mRNA expression of markers for mitochondrial biogenesis, (**e**) typical examples of SM protein levels of NRF2 and TOM20 (n = 3/group), (**f**) quantification of full Western blots of NRF2 and TOM20 (n = 4–5/group), and (**g**) typical electron microscopy examples of m. gastrocnemius (white arrows indicating mitochondria) and quantification of mitochondrial (mito) number per field of 55μm^2^; six high power fields were analysed per animal, (n = 2/group). eNOS endothelial nitric oxide synthase, SIRT1 sirtuin 1, PGC-1α peroxisome proliferator-activated receptor gamma coactivator 1-alpha, NRF2 nuclear respiratory factor 2, TOM20 outer mitochondrial membrane protein. WT wild-type mice, EpoR-tKO EpoR-tissue knock-out mice. Data are presented as mean value ± standard error of the mean (SEM). WT vs. EpoR-tKO: *p < 0.05, **p < 0.01. Raw data with zoomable files at high resolution are accessible at www.nanotomy.org

The generation of new mitochondria, and thereby increasing the mitochondrial content, is known as a process called mitochondrial biogenesis and is regulated by multiple signalling pathways and transcription factors [11, 40]. Interestingly, rhEPO has been previously shown to enhance exercise capacity and stimulate mitochondrial biogenesis through regulation of markers including eNOS and PGC-1α [7]. In our model, mitochondrial content expressed as the mitochondrial to nuclear DNA ratio was unaltered in cardiac muscle as was the mRNA expression of molecular markers for mitochondrial biogenesis (Fig. 2a–b). In skeletal muscle, however, mitochondrial DNA content was reduced by 50% in EpoR-tKO mice compared to WT mice (Fig. 2c). Similarly, protein levels of the outer mitochondrial membrane protein (TOM20) were also significantly lower in skeletal muscle of EpoR-tKO mice (Fig. 2e–f, Supplementary Fig. S2). These reductions in mitochondrial content were associated with similar reductions in the expression of key regulators of mitochondrial biogenesis, eNOS, SIRT1, PGC-1α and NRF2 (Fig. 2d). In addition, protein levels of NRF2, a key regulator of mitochondrial biogenesis, were also decreased in EpoR deficient mice (Fig. 2e–f, Supplementary Fig. S2). To corroborate whether the differences in mitochondrial DNA content and TOM20 protein levels paralleled with similar reductions in the actual number of mitochondria, we next performed electron microscopy (EM) of m. gastrocnemius muscle. Strikingly, quantification of the number of mitochondria in our EM datasets revealed that the number of interfibrillar mitochondria was reduced in skeletal muscle of EpoR-tKO mice (Fig. 2g, available for complete datasets). This was accompanied by a trend towards a decrease in mRNA expression of oxidative muscle fibre types, which may also reflect a reduction in mitochondrial abundance (Supplementary Fig. S3a) [40].

Next, we aimed to determine whether the reductions in mitochondrial content and biogenesis were also associated with enhanced elimination of mitochondria, through a process called mitophagy [11]. We therefore assessed p62, LC3B I and LC3B II at protein level, which are markers for mitophagy. We did not observe significant differences in protein levels of mitophagy markers in skeletal muscle of EpoR-tKO mice compared to WT mice (Supplementary Fig. S4a–b). Finally, to assess whether mitochondrial performance was altered in EpoR-tKO mice, oxygen consumption in isolated mitochondria was assessed. Surprisingly, the changes in mitochondrial content were not accompanied by changes in mitochondrial respiratory capacity (Supplementary Fig. S3b) [57]. Together, these data indicate that the EpoR expressed in skeletal muscle regulates mitochondrial biogenesis, without affecting mitochondrial oxidative capacity or mitophagy.

### Exercise performance is impaired in EpoR-tKO mice despite normal haematocrit levels

**Fig. 3:**
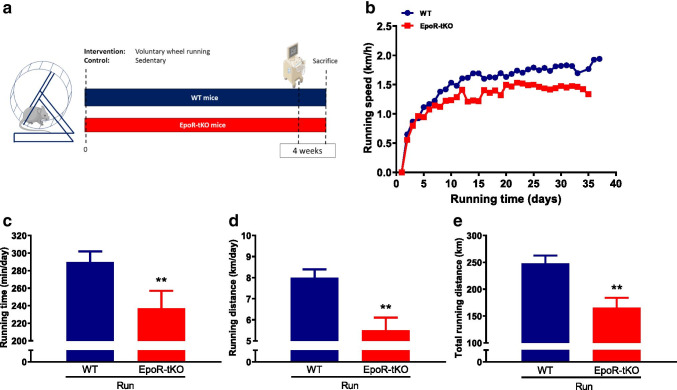
Exercise performance in wild-type and EpoR-tissue knock-out mice. Exercise parameters during 4-week period of voluntary wheel running (n = 11–12/group). Shown are (**a**) experimental design including voluntary wheel running protocol, (**b**) average daily running speed in kilometres per hour (km/h), (**c**) average running time in minutes per day (min/day), (**d**) average daily running distance in kilometres per day (km/day) and (**e**) total running distance in kilometres (km) that mice achieved in the 4-week voluntary wheel running experiment. WT wild-type mice, EpoR-tKO EpoR-tissue knock-out mice, Run running. Data are presented as mean value ± standard error of the mean (SEM). WT vs. EpoR-tKO: **p < 0.01. Part of the illustration in panel **a** contains images from Servier Medical Art by Servier, licensed under a Creative Commons Attribution 3.0 unported license

We next sought to determine whether the reductions in mitochondrial content in skeletal muscle affected exercise performance. For this purpose, WT and EpoR-tKO mice were housed individually in the presence or absence of a running wheel in their cage (Fig. 3a). With preserved erythropoiesis (Supplementary Table S2), EpoR-tKO running mice had a lower average speed compared to WT mice, spent less time on the running wheel and ran significantly fewer daily and kilometres (Fig. 3b–d). Consequently, mice in the EpoR-tKO Run group covered less total distance compared to mice in the WT Run group (Fig. 3e).

### Cardiac adaptation to exercise is impaired in EpoR-tKO mice

#### Cardiac function

**Fig. 4:**
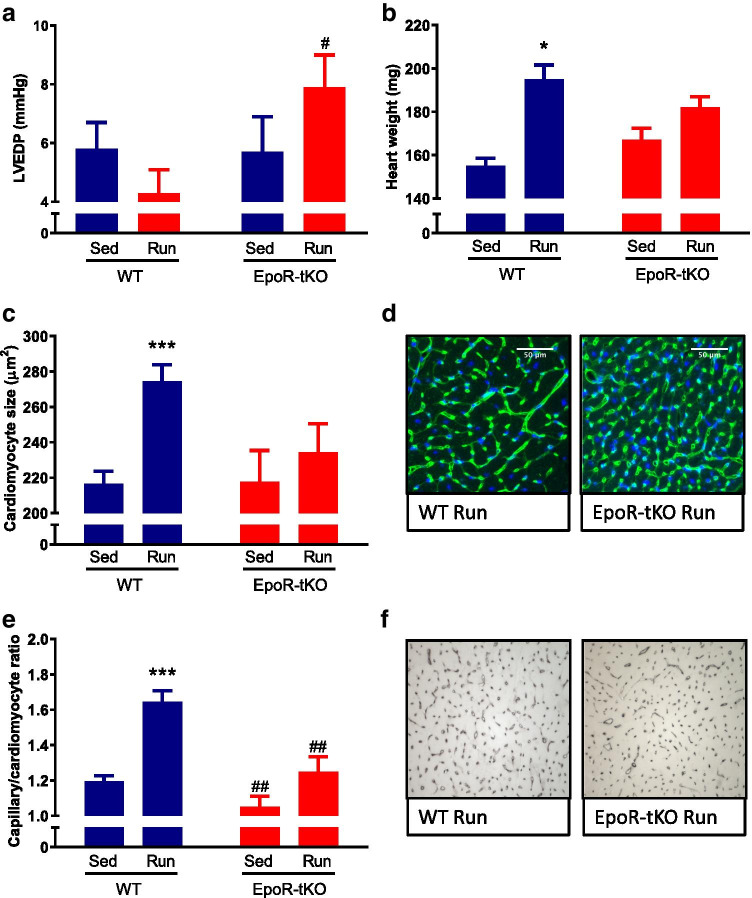
Functional, structural and angiogenic changes in cardiomyocytes: immunohistochemistry analysis. Functional, structural and angiogenic changes in cardiomyocytes after 4 weeks of voluntary wheel running (n = 10–13/group). Shown are (**a**) intra cardiac measurement of left ventricular end-diastolic pressure (LVEDP) in millimetre of mercury (mmHg) and (**b**) heart weight (HW) in milligrams (mg). Panel **c** displays cardiomyocyte cross-sectional area in micrometres-squared (μm^2^) and (**d**) typical examples of cardiomyocyte size. In panel **e**, the capillary-to-cardiomyocyte ratio is shown, together with typical examples of capillary density in panel **f**. WT wild-type mice, EpoR-tKO EpoR-tissue knock-out mice, Sed sedentary, Run running. Data are presented as mean value ± standard error of the mean (SEM). Sed vs. Run: *p < 0.05, ***p < 0.001, Sed vs. Sed and Run vs. Run: # p < 0.05, ## p < 0.01

To determine if loss of EpoR signalling in the cardiac muscle affected the adaptive changes in cardiac performance induced by exercise, intracardiac pressure measurements and echocardiography were performed. Voluntary exercise resulted in a significant improvement in both contractility and relaxation of the left ventricle (dP/dt max, dP/dt min, respectively), as well as a comparable effect in fractional shortening among both groups (Supplementary Table S3). However, left ventricular end-diastolic pressure (LVEDP), mean arterial pressure (MAP) and systolic blood pressure (SBP) were considerably increased in EpoR-tKO mice compared to WT mice after exercise (Fig. 4a, Supplementary Table S3).

#### Cardiac hypertrophy

Exercise resulted in a significant increase in cardiac weight in WT mice (WT Sed 155 ± 3.6 vs. WT Run 195 ± 6.6 mg, p < 0.05) (Fig. 4b). In contrast, exercise did not significantly increase cardiac mass in EpoR-tKO mice (Fig. 4b). Body weight was comparable among the experimental groups (Supplementary Table S2). Accordingly, when cardiac weight was adjusted for body weight, a similar trend was observed (Supplementary Table S2).

Analogous to pathological stress, the cardiac physiological response to sustained exercise includes increasing cardiomyocyte size [44]. Exercise resulted in a 26% increase in cardiomyocyte cross-sectional area (CSA) in WT mice (Fig. 4c–d). In contrast, exercise did not result in a considerable increase in CSA in EpoR-tKO mice (Fig. 4c–d). Similarly, the increase in the mRNA expression of alpha-actinin 1 (ACTA-1), a molecular marker of muscle growth, was more pronounced in WT Run than in the EpoR-tKO Run group (Supplementary Table S4). Furthermore, atrial natriuretic peptide (ANP) expression was increased and a shift towards β-myosin heavy chain (β-MHC) isoforms was observed in the LV of EpoR-tKO Run mice, suggesting that exercise induced subtle maladaptive remodelling in EpoR-tKO mice [32] (Supplementary Table S4).

#### The angiogenic response in cardiac muscle

In response to exercise, cardiomyocyte hypertrophy is paralleled by a corresponding expansion of the capillary network [44]. Cardiac capillary-to-myocyte ratio was slightly, but significantly reduced in sedentary EpoR-tKO mice compared to sedentary WT mice (p < 0.01) (Fig. 4e–f). Furthermore, the normal increase in capillary density in response to exercise that was observed in WT Run mice was abrogated in the EpoR-tKO Run group (Fig. 4e–f). Similarly, the mRNA expression of vascular endothelial growth factor α (VEGF-α), a critical regulator of angiogenesis, was substantially increased in response to exercise in WT mice, but not altered by exercise in EpoR-tKO mice (Supplementary Table S4).

### The adaptive response of skeletal muscle to exercise is impaired in EpoR-tKO mice

#### Myocyte hypertrophy

**Fig. 5:**
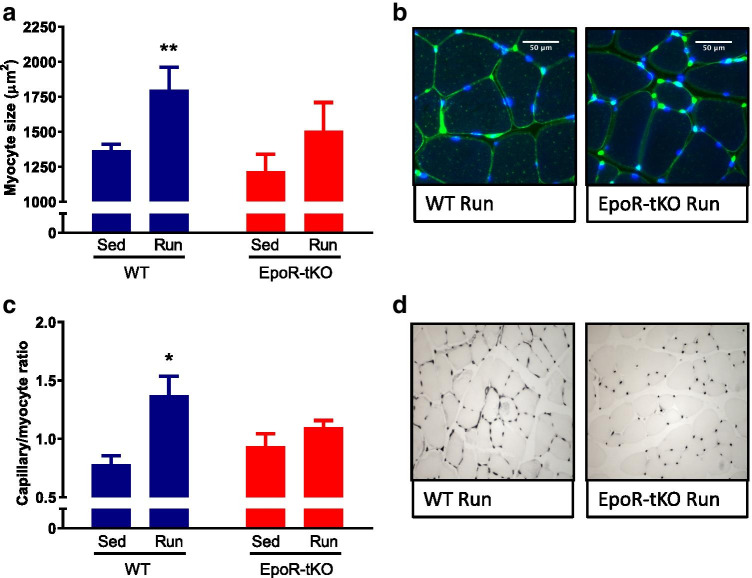
Structural and angiogenic changes in skeletal myocytes: immunohistochemistry analysis. Structural and angiogenic changes in skeletal myocytes after 4 weeks of voluntary wheel running (n = 10–13/group). Shown are (**a**) skeletal myocyte (SM) cross-sectional area (CSA) in micrometres-squared (μm^2^) and (**b**) typical examples of SM size. In panel **c**, the skeletal capillary-to-myocyte ratio is shown, together with typical examples of capillary density in panel **d**. WT wild-type mice, EpoR-tKO EpoR-tissue knock-out mice, Sed sedentary, Run running. Data are presented as mean value ± standard error of the mean (SEM). Sed vs. Run: *p < 0.05, **p < 0.01

Similar to cardiac muscle, skeletal muscle fibres also develop myocyte hypertrophy in response to exercise [40]. Exercise resulted in a 32% increase in myocyte CSA in the WT Run group, but this increase upon exercise was absent in EpoR-tKO mice (Fig. 5a–b). This pattern was similar for the mRNA expression of ACTA-1, and can be observed in Supplementary Table S4.

#### The angiogenic response in the skeletal muscle

Physiological myocyte hypertrophy in skeletal muscle is also paralleled by expansion of the capillary network [40]. To determine whether the EpoR is also critical for adaptive angiogenesis in skeletal muscle, capillary density was evaluated in the m. gastrocnemius. In this skeletal muscle, an abrogated response in capillary/myocyte ratio and VEGF-α mRNA expression was also observed in EpoR-tKO mice (Fig. 5c–d, Supplementary Table S4).

## Discussion

The role of the non-haematopoietic EpoR in the adaptive response to physiological stress remains unexplored. In this study, we aimed to determine the role of the EpoR in cardiac and skeletal muscle in the physiological adaptation to exercise. While EpoR-tKO mice were phenotypically normal, mitochondrial content in skeletal muscle of EpoR-tKO mice was significantly reduced, and mitochondrial biogenesis was impaired compared to wild-type mice. EpoR-tKO mice also displayed reduced exercise performance in response to voluntary wheel running, associated with reductions in myocyte hypertrophy and adaptive angiogenesis in both cardiac and skeletal muscle. Taken together, our findings indicate that the endogenous EPO-EpoR system is critical for basal mitochondrial biogenesis in skeletal muscle. We also show that the associated reductions in mitochondrial content are accompanied by impaired exercise performance, indicating that the extra-haematopoietic EpoR is critical for the physiological adaptation to exercise.

Cardiac and skeletal muscle require considerable plasticity to adapt to continuous variations in demand. The response to endurance exercise training is remarkably similar in cardiac and skeletal muscle, including an increase in myocyte size, expansion of the capillary network, changes in muscle fibre isoforms and an increase in mitochondrial density [40, 43]. While several key regulators of physiological hypertrophy have been identified [32], the role of the endogenous EPO-EpoR system in cardiac and skeletal muscle has not been well described. The tissue-specific EpoR has however been widely studied in pathological conditions and findings from these studies suggest that the EpoR exerts protective effects in response to pathological stress [16, 33, 48]. Current knowledge therefore mainly focusses on the effects of the tissue-specific EpoR in disease but knowledge about its physiological role is limited. The sparse evidence indicating a critical role for this system in the response to exercise was often confounded by the concomitant changes in haematocrit. For instance, mutated mice with systemic EPO-deficiency have reduced exercise performance, associated with oxidative and metabolic stress in skeletal muscle and reduced expression of fast oxidative muscle fibres; yet, these observations were done in the presence of severe anaemia [30]. rhEPO also improves exercise performance and induces mitochondrial biogenesis in cardiac and skeletal muscle of healthy mice through activation of key regulators of physiological hypertrophy, including AKT, eNOS and PGC-1α [7]. Importantly, these mitochondrial effects preceded any changes in haematocrit, suggesting that they were regulated by a tissue-specific receptor. Mitochondrial respiration was also increased in permeabilised skeletal muscle fibres obtained from athletes after prolonged treatment with rhEPO [39]. Overexpression of EPO in mice also activated PGC-1α and increased the proportion of oxidative muscle fibres, whereas the opposite was observed in our mouse model [51]. While these studies clearly indicate that EPO stimulates oxidative metabolism in muscle cells, it is impossible to exclude the possibility that these results were confounded by off target effects of EPO. Our study suggests that the tissue-specific EpoR is a crucial regulator of basal mitochondrial biogenesis as well as structural and metabolic adaptations to exercise. Regarding the difference in capillary density, it should be noted that we cannot reach definitive conclusions about cause and effect. The insufficient angiogenic response could result from loss of EpoR signalling, but could also be a secondary effect related to reduced oxidative muscle fibres.

The first indication suggesting that a functional EPO receptor was present in the cardiac and skeletal muscle dates back almost two decades [6]. While the presence of a functional extra-haematopoietic EPO-receptor was scrutinised at first, studies using comprehensive analysis have firmly established the presence of a functional EPO receptor in muscle cells [35, 42, 52]. The extra-haematopoietic EpoR comprises a distinct isoform of the EpoR, with a distinct pattern of regulation, and heterodimerises with other receptors such as the beta common receptor (βcR) to compose the innate repair receptor (IRR) [8, 10]. The IRR potentially plays a role in repair mechanisms in response to injury and inflammation; however, some controversy has been described regarding the mechanisms of interaction between these receptor isoforms [8, 10]. Yet, these differences between the tissue and haematopoietic EPO receptor suggest that the tissue receptor can be targeted specifically and several compounds are currently under investigation [8, 10]. This would offer the possibility to exploit the beneficial effects of rhEPO for exercise performance without the untoward effects on haematocrit. Whether these isoforms are capable of regulating exercise performance and exercise-induced muscle growth remains unknown.

Literature describes some degree of discrepancy regarding the role of the skeletal muscle EpoR. Some studies observe little to no effects of the EpoR in skeletal muscle whereas other studies suggest a beneficial role for an EpoR in skeletal muscle [20–22, 51]. For instance, EPO administration shows activation of AKT signalling, but this was accompanied by limited effects on protein synthesis in skeletal muscle after exercise [21]. Also, the role of the EpoR in myogenesis was found to be negligible [22]. However, findings that describe the limited role for the EpoR in skeletal muscle adaptations were based on a single EPO injection or in vitro EPO treatment and do not specifically target the EpoR as described above. Contrarily to the above, our results are in line with previous reports that have indeed demonstrated the role of the EpoR in basal mitochondrial muscle metabolism in EpoR-tKO mice [51]. These mechanisms have been attributed to the regulation of AMPK and PGC-1α, which in turn modulate oxidative muscle fibre reprogramming [51]. With our study, we now provide the evidence that the EpoR in skeletal muscle is indeed critical for basal mitochondrial biogenesis. Our data suggests that these changes are associated with reduced exercise capacity and impaired adaptation of cardiac and skeletal muscle to voluntary exercise. Future investigations should be focussed on unravelling the possible underlying mechanisms of these adaptations, such as the regulation of AMPK. Taken together with our findings, the EpoR in skeletal muscle could be a novel target to improve skeletal muscle quality in patients with muscle fatigue, mitochondrial myopathies or heart failure-associated exercise intolerance [13, 38, 53]. Of note, ageing EpoR-tKO mice, mostly pronounced in females, also develop severe obesity and insulin resistance, suggesting that targeting the tissue-specific EPO receptor might also be an effective therapy for diabetes [49].

The mechanistic pathways underlying the cardiac effects as a result of the absent EpoR in non-haematopoietic tissues remain unexplored. In our study, we have observed a rise in left ventricular end diastolic pressure, together with similar effects in mean arterial pressure as well as systolic blood pressure in response to exercise. These functional parameters were accompanied by increments of ANP expression and a shift towards β-myosin heavy chain isoforms. Together, these effects describe a phenotype suiting pathological rather than physiological adaptation. We may argue that these effects are primarily due the loss of the EpoR in cardiac tissue; however, these effects may also be related to secondary effects exerted upon the heart. Our findings suggest the primary role for EpoR in response to physiological stress; unfortunately, it remains questionable whether these effects are in fact primary. Therefore, future studies are required in order to determine the specific underlying mechanisms of the impaired physiological response.

Several limitations of our analysis need to be acknowledged. For instance, this is a model of voluntary wheel running in which the workload on cardiac and skeletal muscle is relatively subtle and the amount of exercise varied between the groups, whereas forced or exhaustive exercise may cause more rigorous and equal effects on cardiac and skeletal muscle. Nevertheless, exercise-induced hypertrophy in our WT mice was substantial and similar studies also demonstrated substantial muscle growth induced by voluntary wheel running models [1, 17, 28]. Nonetheless, the role of the EpoR in the adaptation to more strenuous forms of exercise may also be different. The varied amount of exercise performed, however, can be attributed to our findings of decreased mitochondrial content in skeletal muscle of EpoR-tKO mice, resulting in impaired exercise performance and physiological adaptation. Next to this limitation, it should also be acknowledged that we compared mitochondrial content and molecular markers for mitochondrial biogenesis in our study. From this data, we cannot conclude definitely that the actual generation of new mitochondria was perturbed. Finally, this is a mouse model in which mice were rescued from lethality by erythroid lineage-restricted expression of the EpoR. Therefore, the outcomes cannot be interpreted as for cardiac and skeletal myocyte-specific KO mice.

In conclusion, this study provides evidence that the endogenous EPO-EpoR system controls mitochondrial biogenesis in skeletal muscle. We also provide evidence that reduced mitochondrial biogenesis in EpoR-deficient mice was associated with impaired exercise performance, suggesting that the EpoR expressed in cardiac and skeletal muscle is critical for physiological exercise and its adaptation.

## Supplementary Information

Below is the link to the electronic supplementary material.

Supplementary file1 (DOCX 760 KB)

## Data Availability

The data from the current study are available from the corresponding author on reasonable request.
