# Supplementary material for: The erythropoietin receptor expressed in skeletal muscle is essential for mitochondrial biogenesis and physiological exercise
Source: Pflugers Arch. 2021 Jun 17;473(8):1301–13. doi: 10.1007/s00424-021-02577-4 (PMC8302562; doi:10.1007/s00424-021-02577-4)
Supplement: Supplementary file 1 — Supplementary file1 (DOCX 760 KB) [file 424_2021_2577_MOESM1_ESM.docx]

**Supplementary information**

**The erythropoietin receptor expressed in skeletal muscle is essential for mitochondrial biogenesis and physiological exercise**

Kirsten T. Nijholt, BSc^1^, Laura M.G. Meems, MD, PhD^1^, Willem P.T. Ruifrok, MD, PhD^1^, Alexander H. Maass, MD, PhD^1^, Salva R. Yurista, MD, PhD^1^, Mario G. Pavez-Giani, PhD^1^, Belend Mahmoud, BSc^1^, Anouk H. G. Wolters, BSc^2^, Dirk J. van Veldhuisen, MD, PhD^1^, Wiek H. van Gilst, PhD^1^, Herman H.W. Silljé, PhD^1^, Rudolf A. de Boer, MD, PhD^1^, B. Daan Westenbrink, MD, PhD^1^*

^1^Department of Cardiology, University Medical Centre Groningen, University of Groningen, Groningen, The Netherlands

^2^Department of Cell Biology, University Medical Centre Groningen, University of Groningen, Groningen, The Netherlands

Corresponding author:

Dr. B.D. Westenbrink, MD, PhD

Department of Cardiology, University Medical Centre Groningen

Hanzeplein 1, 9713 GZ Groningen, The Netherlands

P.O. Box 30.001, HPC AB31, 9700 RB Groningen, The Netherlands

Phone: +31 50 361 22 07, email: [b.d.westenbrink@umcg.nl](mailto:r.a.de.boer@umcg.nl)

Short title: extra-haematopoietic EpoR signalling in exercise

**Methods**

**Mitochondrial isolation for respiratory function**

Directly after sacrifice, muscle tissue was used for isolation of mitochondria. The muscle tissue was collected in mitochondrial isolation buffer. Commencing with the isolation process, tissue was cut into smaller pieces. Thereafter, tissue homogenization was performed with a polytron homogenizer. The process was continued by performing different centrifugation steps until the pellet was completed dissolved. Mitochondrial protein quantification was performed using the BCA assay method (Pierce. No. 232250, ThermoFisher, USA).

SeaHorse Flux Analyzer (Agilent Seahorse Bioscience XF96 analyser) was performed to assess mitochondrial respiratory function. Equal amounts of mitochondrial protein (2-4μg) were pipetted into each well of the XF96 microplate (Agilent 100850-001) on ice. Mitochondrial assay solution (MAS) plus substrate were also pipetted to the microplate. This was followed by centrifugation step at 2000g for 20 minutes at 4°C. Then, prewarmed (37°C) 1x MAS, substrate and initial conditions were added to each well. STATE 3 respiration initial conditions included pyruvate (10mM), malate (2μM) and ADP (4mM). Injection protocol included Port A, Oligomycin (2,5 μg/ml), Port B-C FCCP (4μM), Port D Antimycin/rotenone (4μM) as described previously[1].

**Tables**

**Table S1 Primer sequences**

|  | **5’-3’ Forward** | **5’-3’ Reverse** |
| --- | --- | --- |
| 36B4 | AAGCGCGTCCTGGCATTGTC | GCAGCCGCAAATGCAGATGG |
| EpoR | TCCGGTTCTCCTCGCTATCA | GGCGTCCAGGAGCACTACTT |
| ANP | ATGGGCTCCTTCTCCATCAC | TCTACCGGCATCTTCTCCTC |
| α-MHC | GACAACTCCTCCCGCTTTGG | AAGATCACCCGGGACTTCTC |
| β-MHC | TCTGGAGGCCTTTGGCAATG | GATGCCAACTTTCCTGTTGC |
| eNOS | TCCTAACTTGCCCTGCATCC | GGCAGCCAAACACCAAAGTC |
| SIRT1 | GCAGGTTGCGGGAATCCAA | GGCAAGATGCTGTTGCAAA |
| PGC-1α | CGGAAATCATATCCAACCAG | TGAGGACCGCTAGCAAGTTTG |
| NRF2 | ATGGACTTGGAGTTGCCACC | TCTTGCCTCCAAAGGATGTCA |
| HK2 | GCCAGCCTCTCCTGATTTTAGTGT | GGGAACACAAAAGACCTCTTCTGG |
| ND1 | CTAGCAGAAACAAACCGGGC | CCGGCTGCGTATTCTACGTT |
| MHC I | GAGCCTTGGATTCTCAAACG | CTTGCTACCCTCAGGTGGCT |
| MHC IIa | CCATTCAGAGCAAAGATGCAGG | GCATAACGCTCTTTGAGGTTG |
| MHC IIx | AGGACCAAGTGAGTGAGCTG | CTTTTCGTCTAGCTGGCGTGA |
| ACTA-1 | TGCCATGTATGTGGCTATCCA | TCCCCAGAATCCAACACGAT |
| VEGF-α | ACTGGACCCTGGCTTTACTG | CAGTAGCTTCGCTGGTAGAC |

Primer sequences for the following genes: *(1)* 36B4, housekeeping gene; *(2)* EpoR, erythropoietin receptor; *(3)* ANP, atrial natriuretic peptide; *(4)* α-MHC, alpha-myosin heavy chain; *(5)* β-MHC, beta-myosin heavy chain; *(6)* eNOS, endothelial nitric oxide synthase; *(7)* SIRT1, sirtuin 1; *(8)* PGC-1α, peroxisome proliferator-activated receptor gamma coactivator 1 alpha; *(9)* NRF2, nuclear respiratory factor 2; *(10)* HK2, hexokinase 2; *(11)* ND1, NADH dehydrogenase 1; *(12)* MHC I, myosin heavy chain isoform I; *(13)* MHC IIa, myosin heavy chain isoform IIa; *(14)* MHC IIx, myosin heavy chain isoform IIx; *(15)* ACTA-1, actin alpha 1; *(16)* VEGF-α vascular endothelial growth factor alpha.

**Table S2** **Haematocrit levels and clinical characteristics after four weeks of voluntary wheel running**

|  | **WT Sed**  **(N=10)** | **WT Run**  **(N=12)** | **EpoR-tKO Sed (N=13)** | **EpoR-tKO Run**  **(N=11)** |
| --- | --- | --- | --- | --- |
| Ht (%) | 45±1 | 45±1 | 45±2 | 44±1 |
| HW (mg) | 155±3.6 | 195±6.6 * | 167±5.4 | 182±5.0 |
| BW start (gr) | 26.3±0.4 | 26.5±0.4 | 27.5±0.6 | 26.4±0.5 |
| BW sac (gr) | 27.1±0.4 | 27.1±0.4 | 28.1±0.8 | 26.6±0.4 |
| Delta BW (gr) | 0.7±0.3 | 0.8±0.3 | 1.1±0.6 | 0.1±0.3 |
| HW/BW ratio (mg/gr) | 5.7±0.1 | 7.1±0.2 *** | 5.9±0.2 | 6.8±0.2 * |

Data are shown of animals after a four-week period of voluntary wheel running. Values represent means ± standard error of the mean (SEM). Ht= haematocrit, HW= heart weight, BW= body weight, WT= wild type mice, EpoR-tKO= EpoR-tissue knock-out mice, Sed= Sedentary, Run= Running. Sed *vs.* Run: *p<0.05, ***p<0.001.

**Table S3** **Intracardiac and echocardiographic parameters at sacrifice after four weeks voluntary wheel running**

|  | **WT Sed**  **(N=10)** | **WT Run**  **(N=12)** | **EpoR-tKO Sed (N=13)** | **EpoR-tKO Run**  **(N=11)** | |
| --- | --- | --- | --- | --- | --- |
| HR (bpm) | 495±21 | 484±22 | 532±38 | 486±25 |  |
| SBP (mmHg) | 102±2 | 100±2 | 109±4 | 111±3 # |  |
| DBP (mmHg) | 63±2 | 61±2 | 65±4 | 69±3 |  |
| MAP (mmHg) | 76±1 | 74±2 | 80±4 | 82±2 # |  |
| LVEDP (mmHg) | 5.8±0.9 | 4.3±0.8 | 5.7±1.2 | 7.9±1.1 # |  |
| dPdtmax (mmHg/s) | 8969±293 | 10611±366 * | 8901±585 | 11115±572 * |  |
| dPdtmin (mmHg/s) | -8318±484 | -10057±467 * | -8278±413 | -11192±660 * |  |
| IVSs (mm) | 1.23±0.05 | 1.32±0.07 | 1.30±0.06 | 1.41±0.07 |  |
| LVPWs (mm) | 1.08±0.05 | 1.23±0.05 | 1.26±0.06 | 1.34±0.08 |  |
| FS (%) | 29.97±2.37 | 31.68±2.1 | 33.67±1.5 | 35.52±2.1 |  |
| CO (ml/min) | 21.57±1.9 | 28.10±1.6 * | 24.41±1.0 | 23.10±1.8 |  |
| SV (μl) | 62.48±4.1 | 74.00±3.5 | 65.63±2.8 | 64.98±4.6 |  |

Data are shown of animals after a four-week period of voluntary wheel running. Values represent means ± standard error of the mean (SEM). HR= heart rate, SBP= systolic blood pressure, DBP= diastolic blood pressure, MAP= mean arterial pressure, LVEDP= left ventricular end diastolic pressure, dPdtmax and dPdtmin= maximal rates of increase and decrease in left ventricular pressure, IVSs= thickness of the left ventricular septum during systole, LVPWs= thickness of the left ventricular posterior wall during systole, FS= fractional shortening, CO= cardiac output, SV= stroke volume, WT= wild type mice, EpoR-tKO= EpoR-tissue knock-out mice, Sed= Sedentary, Run= Running. Sed *vs.* Run: *p<0.05, Run *vs.* Run: # p<0.05.

**Table S4 Molecular analysis of markers for adaptation in cardiac and skeletal muscle**

|  | **WT Sed**  **(N=10)** | **WT Run**  **(N=12)** | **EpoR-tKO Sed (N=13)** | **EpoR-tKO Run**  **(N=11)** | |
| --- | --- | --- | --- | --- | --- |
| LV ANP | 1.00±0.08 | 1.24±0.10 | 0.79±0.06 | 1.15±0.13* |  |
| LV βMHC/αMHC | 1.00±0.10 | 0.76±0.08* | 0.72±0.12# | 1.04±0.11*# |  |
| LV ACTA-1 | 1.00±0.03 | 2.33±0.27*** | 0.93±0.08 | 1.58±0.14** |  |
| LV VEGF-α | 1.00±0.02 | 1.27±0.10* | 1.10±0.09 | 1.08±0.10 |  |
| SM ACTA-1 | 1.00±0.04 | 0.93±0.06 | 1.00±0.05 | 0.84±0.05** |  |
| SM VEGF-α | 1.00±0.04 | 1.09±0.08 | 0.87±0.08## | 0.86±0.07## |  |

Data are shown of animals after a four-week period of voluntary wheel running. Values represent means ± standard error of the mean (SEM). LV= left ventricular, SM= skeletal muscle, ACTA-1= actin alpha 1, VEGF-α= vascular endothelial growth factor alpha, WT= wild type mice, EpoR-tKO= EpoR-tissue knock-out mice, Sed= Sedentary, Run= Running. Sed *vs.* Run: *p<0.05, **p<0.01, ***p<0.001, Sed *vs.* Sed and Run *vs.* Run: # p<0.05, ## p<0.01.

**Figures**

**Fig. S1 Phenotyping of tissue-specific erythropoietin receptor deficient (EpoR-tKO) mice**

Phenotyping of tissue-specific erythropoietin receptor deficient (EpoR-tKO) mice, (n= 11-13/group). Shown are *(a)* haematocrit (Ht) levels (%) and *(b)* left ventricular (LV) mRNA expression of cardiac markers. ANP= atrial natriuretic peptide, BNP= brain natriuretic peptide, α-MHC= alpha-myosin heavy chain, β-MHC= beta-myosin heavy chain, WT= wild type mice, EpoR-tKO= EpoR-tissue knock-out mice. Data are presented as mean value ± standard error of the mean (SEM).

**Fig. S2 Mitochondrial biogenesis and content in basal skeletal muscle of wild type and EpoR-tKO mice**

Markers for mitochondrial biogenesis and content in skeletal muscle (SM) at baseline. Shown are, *(a)* full western blots expressing proteins associated with mitochondrial biogenesis and content (n= 4-5/group). NRF2= nuclear respiratory factor 2, TOM20= outer mitochondrial membrane protein TOM20, GAPDH= glyceraldehyde 3-phosphate dehydrogenase. WT= wild type mice, EpoR-tKO= EpoR-tissue knock-out mice.

**Fig. S3. Mitochondrial respiratory capacity and skeletal muscle myosin heavy chain isoforms of wild type and EpoR-tKO mice at baseline**

Basal phenotyping of skeletal muscle (SM) fibre types and mitochondrial function. Shown are *(a)* mRNA expression of myosin heavy chain (MHC) isoforms in skeletal muscle (n=12/group) and *(b)* mitochondrial state 3 respiration in muscle (n= 3/group). WT= wild type mice, EpoR-tKO= EpoR-tissue knock-out mice. Data are presented as mean value ± standard error of the mean (SEM).

**Fig. S4 Mitophagy in basal skeletal muscle of wild type and EpoR-tKO mice**

Parameters for mitophagy in skeletal muscle (SM) at baseline. Shown are, *(a)* SM protein levels of markers for mitophagy (n= 4-5/group) and *(b)* typical examples of western blots expressing proteins associated with mitophagy. P62= ubiquitin protein 62, LC3B= light chain 3B, GAPDH= glyceraldehyde 3-phosphate dehydrogenase. WT= wild type mice, EpoR-tKO= EpoR-tissue knock-out mice. Data are presented as mean value ± standard error of the mean (SEM).

**References**

1. Yu H, Tigchelaar W, Koonen DPY, Patel HH, de Boer RA, van Gilst WH, Westenbrink BD, Silljé HHW (2013) AKIP1 Expression Modulates Mitochondrial Function in Rat Neonatal Cardiomyocytes. PLoS One 8:e80815. doi: 10.1371/journal.pone.0080815
